# Nitrosothiol-Trapping-Based Proteomic Analysis of S-Nitrosylation in Human Lung Carcinoma Cells

**DOI:** 10.1371/journal.pone.0169862

**Published:** 2017-01-12

**Authors:** Shani Ben-Lulu, Tamar Ziv, Pnina Weisman-Shomer, Moran Benhar

**Affiliations:** 1 Smoler Proteomics Center and Faculty of Biology, Technion-Israel Institute of Technology, Haifa, Israel; 2 Department of Biochemistry, Rappaport Faculty of Medicine, Technion-Israel Institute of Technology, Haifa, Israel; Albany Medical College, UNITED STATES

## Abstract

Nitrosylation of cysteines residues (S-nitrosylation) mediates many of the cellular effects of nitric oxide in normal and diseased cells. Recent research indicates that S-nitrosylation of certain proteins could play a role in tumor progression and responsiveness to therapy. However, the protein targets of S-nitrosylation in cancer cells remain largely unidentified. In this study, we used our recently developed nitrosothiol trapping approach to explore the nitrosoproteome of human A549 lung carcinoma cells treated with S-nitrosocysteine or pro-inflammatory cytokines. Using this approach, we identified about 300 putative nitrosylation targets in S-nitrosocysteine-treated A549 cells and approximately 400 targets in cytokine-stimulated cells. Among the more than 500 proteins identified in the two screens, the majority represent novel targets of S-nitrosylation, as revealed by comparison with publicly available nitrosoproteomic data. By coupling the trapping procedure with differential thiol labeling, we identified nearly 300 potential nitrosylation sites in about 150 proteins. The proteomic results were validated for several proteins by an independent approach. Bioinformatic analysis highlighted important cellular pathways that are targeted by S-nitrosylation, notably, cell cycle and inflammatory signaling. Taken together, our results identify new molecular targets of nitric oxide in lung cancer cells and suggest that S-nitrosylation may regulate signaling pathways that are critically involved in lung cancer progression.

## Introduction

Nitric oxide (NO) is a versatile and ubiquitous signaling molecule that regulates diverse physiological and pathological processes. Substantial evidence links NO to cancer development and progression, however, the role of NO in cancer is multifaceted and complex, exerting both pro- and anti-tumor effects [1–4]. This complexity stems from the multitude of cellular processes that are influenced by NO in the tumor, its microenvironment and in the immune system. At present, there is insufficient understanding regarding the role of NO in tumor progression or suppression.

The physiological and pathological functions of NO are substantially mediated by S-nitrosylation, the covalent attachment of a nitroso group to a cysteine thiol to form an S-nitrosothiol (SNO) [5, 6]. A role for S-nitrosylation is cancer has recently began to emerge [7–9]. For example, nitrosylation of several oncoproteins, including epidermal growth factor receptor (EGFR), Src and H-Ras, has been proposed to exert tumor-promoting effects [10, 11]. Further, it has been demonstrated that elevated S-nitrosylation in mice, caused by genetic ablation of S-nitrosoglutathione reductase, promotes hepatocarcinogenesis [12]. Conversely, nitrosylation of the androgen receptor may act to negatively regulate prostate tumor growth [13]. Likewise, inhibitory S-nitrosylation of other pro-inflammatory and pro-oncogenic proteins, such as NF-κB[14], STAT3[15] and MEK1[16] is expected to exert anti-inflammatory and anti-tumor effects. Although SNO-based regulation of cancer-related proteins is increasingly recognized, there is still limited information on the complement of cancer cell proteins affected by S-nitrosylation, thus hampering the understanding of the role of S-nitrosylation in tumor progression [9].

Recent years have witnessed significant progress in the development of analytical tools for proteome-wide analysis of S-nitrosylation. In particular, the biotin-switch method and variations thereof have enabled the proteomic analysis of S-nitrosylation in multiples cells, tissues, organisms, and disease states [17–20]. However, to date, only a few studies have explored the nitrosoproteome of cancer cells [21–23]. Recently, we have developed a novel proteomic approach to identify nitrosylated proteins based on SNO trapping by the redox protein thioredoxin (Trx) [16]. Trx has been shown to reduce SNOs using its pair of active-site cysteines, Cys32 and Cys35 (human Trx numbering), which function as the catalytic and resolving cysteines [24, 25]. A Trx mutant that lacks the resolving cysteine, Trx(C35S), can trap SNO substrates in a mixed disulfide complex [16]. By coupling SNO trapping by Trx(C35S) with mass spectrometry (MS)-based proteomics we identified a large number of SNO proteins in monocytes and macrophages and uncovered potential new roles for S-nitrosylation in the regulation of macrophage function [16].

The goal of the present study was to begin to characterize the nitrosoproteome of lung cancer cells. Lung cancer is the leading cause of cancer deaths worldwide. Due to its unique structure, the lung is vulnerable to numerous pollutants, gases, oxidants and toxicants. NO has been implicated in the development of lung cancer, which is commonly associated with tobacco use, exposure to chemical irritants, lung infection and inflammation [26]. Consistent with this are observations of increased levels of exhaled NO in lung cancer patients, and elevated inducible NO synthase (iNOS) expression in lung tumor cells, alveolar and tumor-associated macrophages, pulmonary endothelium, and airway epithelium in these patients [27]. Moreover, genetic ablation of iNOS has been shown to reduce lung tumor growth in a mouse model [28]. Accumulating evidence indicates that nitrosylation regulates several proteins involved in lung tumorigenesis, such as Ras and EGFR [29, 30]. However, a large-scale analysis of S-nitrosylation in the context of lung cancer has not been reported yet.

In this work, we used SNO trapping by Trx for large-scale analysis of S-nitrosylation in human A549 lung cancer cells. Using this approach we report the identification of hundreds of candidate nitrosylated proteins, most of which represent novel SNO targets. The present findings suggest new potential roles for S-nitrosylation in regulating lung cancer cell growth, homeostasis, and survival.

## Materials and Methods

### Reagents

Cytokines were obtained from Peprotech (Rocky Hill, NJ). S-Nitrosocysteine (CysNO) was synthesized by combining an equimolar concentration of L-cysteine with sodium nitrite in 0.2 N HCl and used within 1 h. We obtained antibodies against the following: galectin 1 (ab25138), NEDD4 (ab14592) and serpin B6 (ab97330) from Abcam (Cambridge, MA) (respective catalog numbers in parentheses). Tissue culture media and reagents were from Biological Industries (Beit Haemek, Israel). His-tagged proteins Trx(C35S) was expressed and purified from *Escherichia coli* as previously described [16]. Other materials were obtained from Sigma unless otherwise indicated.

### Cell culture and treatment

Human A549 cells, obtained from American Type Culture Collection (ATCC, Manassas, VA), were maintained in Dulbecco’s modified Eagle’s medium (DMEM) supplemented with 10% fetal bovine serum and 1% penicillin/streptomycin at 37°C under 5% CO_2_. To induce S-nitrosylation, exponentially growing A549 cells in a 100-mm Petri dish were treated for 10 min with 500 μM CysNO. S-nitrosylation was also induced by cytokine stimulation. For this purpose, cells were serum starved for 24 h and then stimulated for 72 h with or without a cytokine mix that included lipopolysaccharide (LPS, 0.5 mg/ml), tumor necrosis factor (TNFα, 20 ng/ml), interferon-γ (IFN-γ, 10 ng/ml) and interleukin 1β (IL-1β, 10 ng/ml). During cytokine stimulation 0.1 mM L-arginine was added to the culture medium. Accumulation of nitrite in the cell medium was measured using the Griess reaction. Cell viability was assessed using nonradioactive cytotoxic lactate dehydrogenase (LDH) kit (Promega).

### Thioredoxin-based substrate trapping

The trapping procedure was performed essentially as previously described [16]. Briefly, after various treatments, A549 cells were harvested and permeabilized by incubation with 0.01% digitonin in Tris-buffered saline (TBS, pH 7.5) for 10 min at 4°C on a rotating wheel, followed by centrifugation for 15 min at 4°C to clear the lysate. In parallel, streptavidin agarose beads (Thermo Scientific) were loaded with recombinant Trx proteins (50 μg) in the presence of dithiothreitol (DTT, 20 mM) for 1 h at 4°C and subsequently washed to remove the reductant. The beads were then incubated with supernatants of digitonin-permeabilized cells (approximately 3 mg protein) for 1 h at 4°C. The trapping reaction was quenched with N-ethylmaleimide (NEM, 100 mM) for 15 min at room temperature and thereafter the beads were washed extensively at 4°C as follows: twice in TBS containing 1% Triton X-100, 10 mM NEM, and 1 M NaCl (pH 7.5), once with TBS containing 1% Triton X-100 (pH 7.5), 10 mM NEM, once with TBS containing 0.1% Triton X-100 (pH 7.5), and three times with TBS (pH 7.5). Trapped proteins were eluted with 20 mM DTT in TBS (pH 7.5) for 30 min at room temperature, and then treated with 80 mM iodoacetamide (IAM) for 30 min. Eluted proteins were analyzed by SDS-PAGE. Gels were stained with Krypton Infrared Protein Stain (Pierce) and proteins visualized using the Odyssey infrared imaging system (LICOR Biosciences). For proteomic analysis the above procedure was scaled-up 4-fold.

### Sample preparation and mass spectrometry analysis

Streptavidin agarose pull-down samples representing equal amounts of starting material were separated by a short SDS-PAGE run and the gel was subsequently stained with Coomassie blue. The protein bands were excised from the stained gels, digested with modified trypsin (Promega) in 10% acetonitrile and 10 mM ammonium bicarbonate at a 1:10 enzyme to substrate ratio overnight at 37°C. For analysis of A549 lysate proteome, 10 μg aliquots of the digitonin lysates were treated with 8 M urea and 2.8 mM DTT for 30 min at 60°C and then modified with 8.8 mM IAM for 30 min at room temperature in the dark. The protein sample was digested overnight at 37°C in 2 M urea, 25 mM ammonium bicarbonate with modified trypsin (1:50 enzyme to substrate ratio). A second trypsin digestion was performed for 4 h. The resulting tryptic peptides (from either pull-down or lysate samples) were analyzed by liquid chromatography coupled with tandem mass spectrometry (LC-MS/MS) using a Q Exactive plus mass spectrometer (Thermo Fisher Scientific) fitted with a capillary HPLC (easy nLC 1000, Thermo). The peptides were loaded onto a C18 trap column (0.3 x 5 mm, LC-Packings) connected on-line to a homemade capillary column (75 μm x 20 cm) packed with Reprosil C18-Aqua (Dr Maisch GmbH, Germany) in solvent A (0.1% formic acid in water). The peptide mixture was resolved by a (5 to 28%) linear gradient of solvent B (95% acetonitrile with 0.1% formic acid) for 180 minutes followed by a 5 minute gradient of 28 to 95% and 25 minutes at 95% acetonitrile with 0.1% formic acid in water at flow rates of 0.15 μl/min. Mass spectrometry was performed in a positive mode (m/z 350–1800, resolution 70,000) using repetitively full MS scan followed by collision induces dissociation (HCD, at 35 normalized collision energy) of the 10 most dominant ions (>1 charges) selected from the first MS scan. The AGC settings were 3x10^6^ for the full MS and 1x10^5^ for the MS/MS scans. The intensity threshold for triggering MS/MS analysis was 1x10^4^. A dynamic exclusion list was enabled with exclusion duration of 20 s.

### Data analysis

The MS raw data was analyzed by the MaxQuant software (version 1.4.1.2, http://www.maxquant.org) [31] for peak picking and quantitation, followed by identification using the Andromeda search engine, searching against the human UniProt database (release February 2014, 68949 entries) with mass tolerance of 20 ppm for the precursor masses and for the fragment ions. Methionine oxidation, N-ethylmaleimide on cysteine, carbamidomethyl on cysteine, N-ethylmaleimide+H_2_O on cysteine or lysine and protein N-terminus acetylation were set as variable post-translational modifications. Minimal peptide length was set to six amino acids and a maximum of two miscleavages was allowed. Peptide- and protein-level false discovery rates (FDRs) were filtered to 1% using the target-decoy strategy. Protein table were filtered to eliminate the identifications from the reverse database and from common contaminants. The MaxQuant software was used for label-free semi-quantitative analysis, based on extracted ion currents (XICs) of peptides enabling quantitation from each LC/MS run for each peptide identified in any of the experiments [32]. Only proteins that were identified with at least two peptides in one of the samples are listed in S1 Table. The missing intensity values (those below background levels) were replaced with a minimal value of 10000. Maximum ratio (between sample intensities) was limited to 100 and higher ratios were replaced with 100. The obtained values were log2 transformed. Proteins were considered to represent putative nitrosylation targets if their corresponding log2 ratio was >2 when comparing the CysNO or cytokine samples to their respective controls, in both replicate experiments. To assess functional enrichment, the lists of putative nitrosylation targets were submitted to GeneCodis [33], a web-based tool for the ontological analysis of large lists of genes/proteins. For this analysis, the KEGG pathways analysis was selected. “Fold enrichment” was defined as the number of proteins detected in the sample compared to the total number of proteins expected in the human proteome for each KEGG pathway. The obtained fold-enrichment values were normalized according to the enrichment of each pathway in the lysate proteome, with non-enriched pathways assigned a fold-enrichment of one. Protein interaction network was generated using the STRING database version 9.1 [34]. Interactions were filtered for highest confidence (>0.900) using Experiments, Databases and Text Mining.

### Detection of protein S-nitrosylation with the biotin-switch technique

Detection of endogenously S-nitrosylated proteins was performed with the biotin-switch technique [17, 35] with some modifications. In brief, cells were lysed in lysis buffer (50 mm Hepes, 1% Nonidet P-40, 150 mm NaCl, 1 mm EDTA, 0.1 mm diethylenetriamine pentaacetate, 50 mm NEM, with protease inhibitors, pH 7.5). Cell debris was removed by centrifugation at 13,000g for 15 min at 4°C. A total of 6 mg of protein was used for each experimental condition. Thiol blocking with 20 mm NEM was performed for 30 min at 37°C with frequent vortexing. Proteins were then precipitated with 3 volumes of acetone at −20°C for 30 min. After centrifugation, the protein pellet was washed with cold acetone to remove residual NEM. Pellets were then resuspended in HENS buffer (HEN with 1% SDS), and freshly prepared biotin-HPDP and sodium ascorbate were added to give final concentrations of 0.3 mg/ml and 40 mM, respectively. After 1 h incubation samples were subjected to a second acetone precipitation step and then to streptavidin pull-down followed by Western blot analysis using specific antibodies. Protein bands were detected and quantified with the Odyssey system.

## Results

### Thioredoxin-based substrate trapping coupled to mass-spectrometry-based proteomics identifies candidate nitrosylated proteins in A549 cells

**Fig 1:**
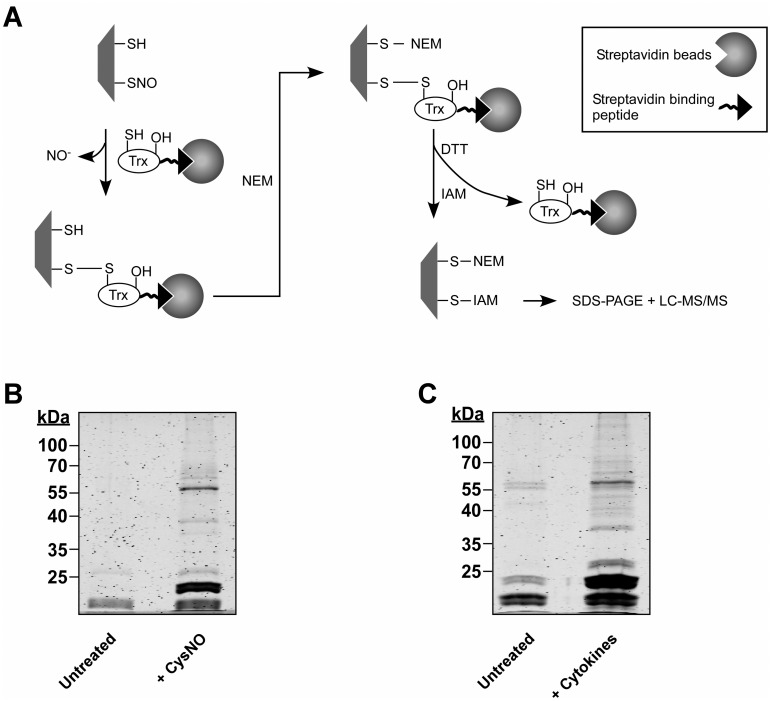
SNO trapping-based analysis of S-nitrosylation in A549 cells. **(**A) Schematic of the proteomic approach. Digitonin cell lysates, obtained from A549 treated with NO donor or with cytokines are incubated with a thioredoxin (Trx) trap mutant, Trx(C35S). In the trap mutant the resolving cysteine is replaced by serine (-OH). The protein also contains a streptavidin binding peptide. Trx(C35S) forms mixed disulfide bonds with nitrosylated substrates and the resulting complexes are pulled-down using avidin agarose. Identification of nitrosylation sites is assisted by differential thiol labeling, involving the sequential application of N-ethylmaleimide (NEM) and iodoacetamide (IAM). Proteins captured in the Trx pull-down are analyzed by SDS-PAGE or liquid chromatography-tandem mass spectrometry (LC-MS/MS). (B) A549 cells were treated with or without 500 μM S-nitrosocysteine (CysNO) for 10 min and thereafter digitonin lysates were incubated with Trx(C35S). Proteins captured by Trx were released by DTT and then analyzed by SDS-PAGE. Gels were stained with Krypton fluorescent protein stain and visualized using the Odyssey infrared imaging system. (C) A549 cells were treated for 72 h with LPS (0.5 mg/ml) and a cytokine mixture that included TNFα (20 ng/ml), IFN-γ (10 ng/ml) and IL-1β (10 ng/ml). Trx-based trapping of nitrosylated proteins was performed as in B.

To initiate the analysis of the lung cancer nitrosoproteome we induced protein S-nitrosylation in A549 cells by treatment with the nitrosylating agent S-nitrosocysteine (CysNO). After cell treatment, digitonin lysates were prepared and subjected to the trapping procedure as illustrated in Fig 1A. Proteins trapped by Trx were pulled down and then identified by liquid chromatography coupled with tandem mass spectrometry (LC-MS/MS). In addition to identification of SNO proteins, we also wished to extract information on individual nitrosylation sites within identified proteins. To accomplish this we used differential thiol labeling (Fig 1A). Briefly, subsequent to the formation of disulfide adducts between trapped proteins and Trx, free thiols were blocked with N-ethylmaleimide (NEM) and then, after the proteins were released from Trx by DTT treatment, nascent thiols we labeled with iodoacetamide (IAM). As such, protein thiols modified by IAM represent putative nitrosylation sites.

Results of trapping experiments performed as described above showed that many proteins were pulled down by Trx(C35S) from CysNO-treated A549 cells whereas much fewer proteins were trapped from untreated cells (Fig 1B). For proteomic analysis, two large-scale experiments were performed and the trapped proteins were subjected to identification by LC-MS/MS using nano-LC coupled to a Q Exactive mass spectrometer. The MS analysis resulted in the identification of 821 proteins with at least two distinct peptides and a false discovery rate (FDR) below 1%, with 534 proteins being identified in both experiments (Sheet A in S1 Table). Only proteins that were identified in both experiments (biological repeats) were considered for further evaluation. We designated proteins as putative SNO targets if their trapping by Trx was increased by CysNO treatment and we employed a semi-quantitative analysis to determine CysNO-dependency (see Materials and Methods). Based on this analysis and using a threshold of 2-fold change (log 2), trapping of 391 proteins was determined to be CysNO-dependent. These 391 proteins thus represent candidate nitrosylated proteins (Sheet A in S1 Table, shaded rows). Focusing on cysteine-containing peptides, we identified 765 peptides that contained cysteines labeled with either NEM or IAM (Sheet B in S1 Table). We applied stringent criteria for SNO site determination. Specifically, only cysteines that were modified by IAM in a CysNO-dependent manner (> 2-fold increase) in both replicate experiments were classified as potential SNO sites. By these criteria, 91 cysteines within 55 proteins were identified as putative SNO sites (Sheet B in S1 Table and see below).

Next, we were interested to analyze protein S-nitrosylation induced by endogenously generated NO. It is known that stimulation of human lung epithelial cells (such as A549 cells) with proinflammatory cytokines induces significant and sustained NO production, mostly via iNOS [36–38]. Based on this information, we treated A549 cells with LPS and a combination of TNF-α, IL-1β and IFN-γ (see Materials and Methods), thereby mimicking a pro-inflammatory environment. In line with previous studies [36–38], we observed a cytokine-dependent NO production, which persisted for at least 3 days, as measured by the accumulation of nitrite in the cell culture medium (S1A Fig). This increased NO synthesis was largely prevented by co-treatment of cells with the iNOS inhibitor 1400W (S1B Fig). In further agreement with previous observations [39], we found that cytokine treatment led to significant cell injury, as determined by measurement of lactate dehydrogenase (LDH) leakage. This cytotoxic effect was however unaffected by treatment with 1400W (S1B Fig). Based on these data, we proceeded to analyze S-nitrosylation after prolonged (72 h) cytokine exposure. By applying the trapping procedure to untreated and cytokine-stimulated A549 cells we found that Trx(C35S) trapped a large number of proteins from the stimulated cells and relatively few proteins from the control (untreated) cells (Fig 1C). Pull-down samples from two replicate experiments were subjected to MS analysis as described above for the CysNO experiments. This analysis identified 869 proteins among which 709 proteins were identified in both experiments (Sheet C in S1 Table). As with the CysNO data set, semi-quantitative analysis was performed for the cytokine data set, which revealed that trapping of 313 proteins was cytokine dependent. We considered these proteins to represent putative nitrosylation targets (Sheet C in S1 Table, shaded rows). By assessing IAM labeling (as above) 201 cysteines belonging to 104 proteins were identified as potential SNO sites (Sheet D in S1 Table and see below). Comparison of the CysNO and cytokine data sets revealed that 504 proteins were common to both sets (Sheet E in S1 Table); of those, trapping of 158 was induced by both CysNO and cytokine treatment (Sheet E in S1 Table, shaded rows). This group of proteins might represent endogenous substrates highly susceptible to undergo S-nitrosylation.

Prolonged cytokine treatment is likely to affect the expression of multiple proteins, which could bias the analysis of the nitrosoproteome. Therefore, in addition to the Trx pull-down samples we also subjected aliquots of the digitonin lysates to protein identification by MS. A triplicate analysis resulted in the identification of 2919 proteins (Sheet F in S1 Table). Semi-quantitative analysis revealed that cytokine treatment resulted in the upregulation of 124 proteins and downregulation of 342 proteins. Rows G-I in Sheet F of S1 Table display the calculated “cytokines/control” ratio for each experiment (green or red represent up- or down-regulation, respectively). Note that these ratios are also displayed next to the Trx pulldown data in Sheet C of S1 Table (brown-colored rows) wherever applicable (530 out of 869 proteins). Accordingly, for most proteins, one can assess whether and to what extent cytokine-dependent changes in the pull-down samples relate to alterations in total protein levels.

### Analysis and validation of the proteomic data

We subjected our lists of candidate SNO targets to bioinformatic analyses in order to compare the present results with previous nitrosoproteome studies and to identify cellular processes and pathways that could be influenced by S-nitrosylation in lung cancer cells. First, we compared our CysNO and cytokine data sets to dbSNO 2.0 (http://140.138.144.145/~dbSNO/index.php) a comprehensive resource of S-nitrosylated proteins from various organisms [40]. dbSNO lists a total of 720 human nitrosylated proteins. Of these 116 proteins were included in the CysNO data set and 46 in the cytokine data set (Sheet G in S1 Table). The comparison between our MS results and dbSNO indicated that the majority of the proteins identified in the present analysis (~ 70%) represent novel nitrosylation targets.

As noted above, we identified as putative SNO sites 91 cysteines (within 55 proteins) in the CysNO data set and 201 cysteines (within 104 proteins) in the cytokine data set (Sheet B and D in S1 Table). Among these proteins, 36 proteins (containing a total of 265 cysteines) are listed in dbSNO. In this set of 36 proteins, we identified 71 cysteines as potential SNO sites, of which 34 cysteines are annotated as such in dbSNO (Sheet H in S1 Table). These findings validate our differential thiol labeling approach (when coupled to Trx-based trapping) for mapping bona fide nitrosylation sites.

**Fig 2:**
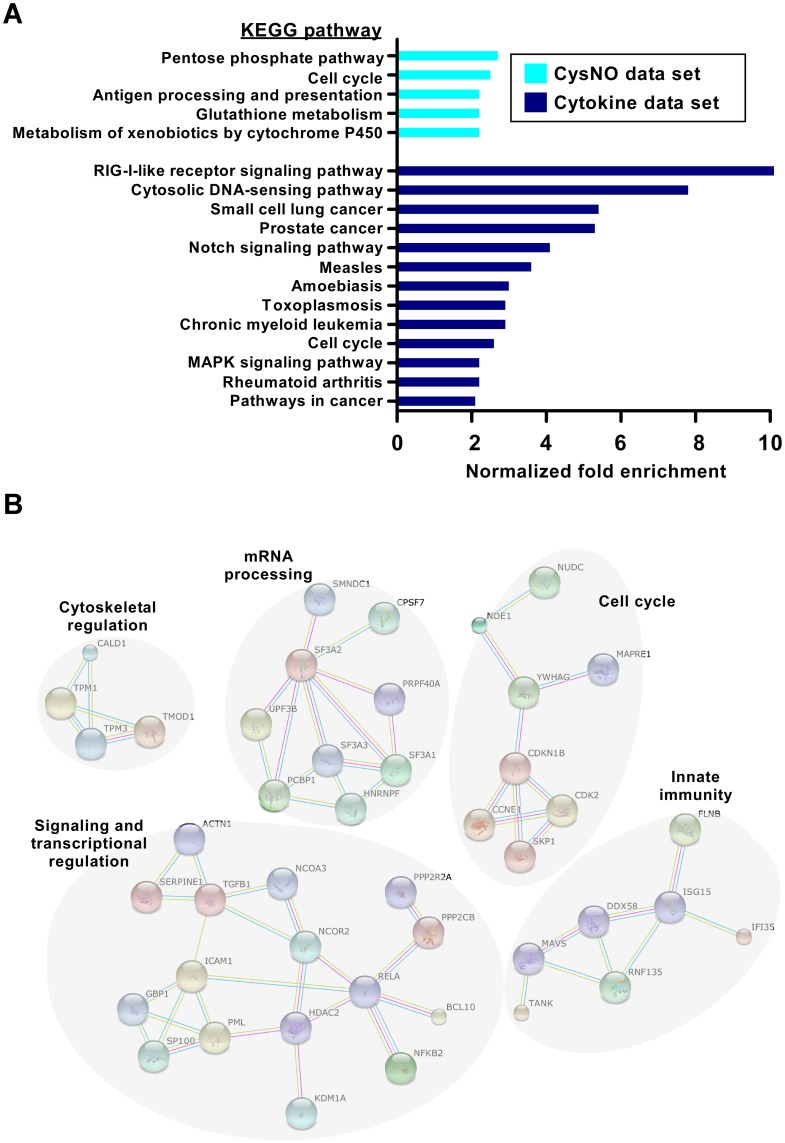
KEGG pathway enrichment and protein interaction analysis of S-nitrosylation in A549 cells. **(**A) Overrepresented KEGG pathways among the identified candidate SNO proteins, ranked by the normalized enrichment fold (see Materials and Methods for details). Proteins were classified into different categories based on KEGG annotations and using the Genecodis algorithm (see also Sheet I in S1 Table). (B) SNO proteins identified in the cytokine data set tend to be functionally related based on protein interaction networks. The diagram shows all high-confidence protein-protein interactions from the STRING interaction database [34].

To obtain functional information for the identified proteins, we submitted the lists of putative SNO targets to Kyoto Encyclopedia of Genes and Genomes (KEGG) pathway enrichment analysis [41] using GeneCodis program [33]. The same enrichment analysis was also applied to the lysate data set (representing the ‘total’ proteome). This allowed us to normalize the calculated fold-enrichment value of each KEGG pathway in the nitrosoproteome to the corresponding fold-enrichment in the total proteome. Using this approach we identified five KEGG pathways as being significantly enriched in the CysNO data set and nine enriched pathways in the cytokine data set (> 2-fold enrichment, FDR corrected p value < 0.01, hypergeometric test; Fig 2A and Sheet I in S1 Table). Notably, this analysis revealed that distinct pathways are overrepresented in the two data sets, with the exception of “cell cycle” that was enriched in both. Of particular interest, several inflammation and cancer cell signaling pathways were enriched in the cytokine data set, suggesting extensive SNO-based regulation of these pathways in the lung cellular inflammatory response. We also analyzed the cytokine data set using the STRING database of known and predicted protein-protein interactions [34]. This analysis showed that cytokine-induced nitrosylation targeted proteins that group into related clusters (Fig 2B), the largest of which is composed of proteins involved in signaling and transcriptional regulation. Additional clusters include proteins involved in mRNA processing, cell cycle, innate immunity and cytoskeletal regulation (Fig 2B).

**Fig 3:**
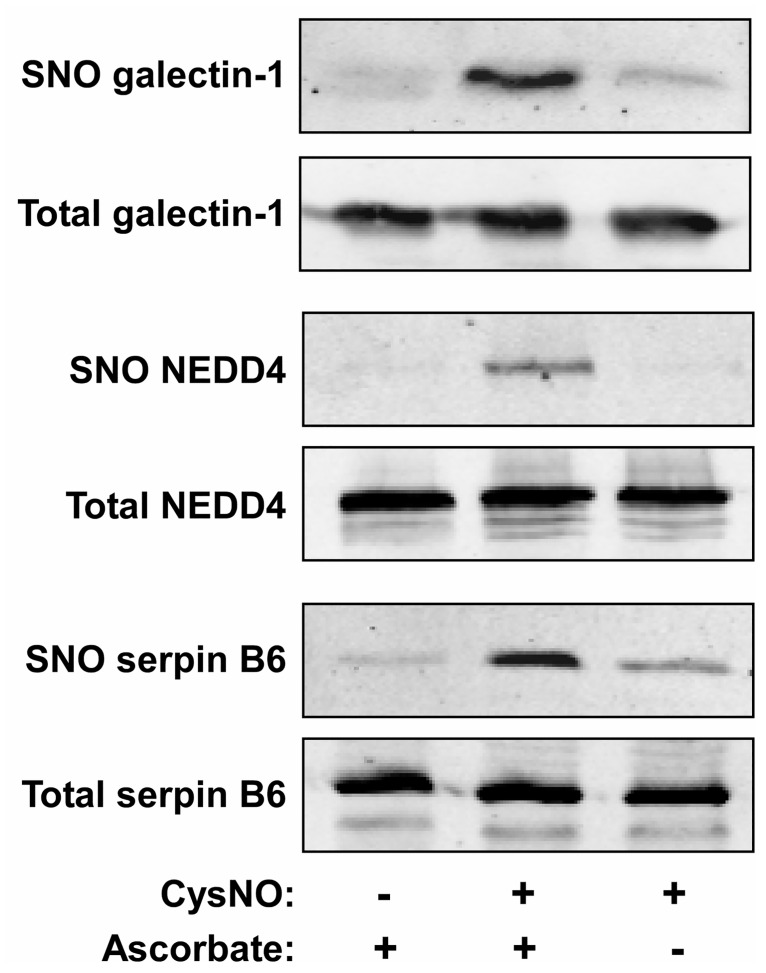
Validation of the proteomic results for selected proteins. A549 cells were treated with or without the CysNO (500 μM; 10 min). The nitrosylation level of galectin-1, NEDD4 and serpin B6 was determined using the biotin-switch assay in the presence or absence of ascorbate.

Finally, to verify the results of the proteomic analysis we analyzed the nitrosylation state of selected proteins in A549 cells. We focused on three candidates: galectin-1, NEDD4, and serpin B6. As shown in Fig 3, a biotin-switch assay showed that all three proteins became nitrosylated in CysNO-treated A549 cells. The biotin-switch assay specificity was confirmed by the observation that omission of ascorbate in the assay system nearly eliminated protein biotinylation. These data suggest that galectin-1, NEDD4, and serpin B6 can undergo S-nitrosylation in A549 cells, and more generally, support the validity of the proteomic results.

## Discussion

S-Nitrosylation is a ubiquitous post-translational modification that regulates a broad spectrum of biological processes. Recent studies have provided growing evidence that S-nitrosylation can influence multiple aspects of cancer cell phenotypes, such as proliferation, migration, DNA repair and apoptosis [7–9]. However, the full complement of SNO targets in most types of cancer cells remains unknown, limiting further biochemical and functional characterization.

In this study, we employed our recently developed SNO trapping approach to identify a large number of candidate nitrosylation targets in A549 human lung cancer cells. We used two experimental conditions to induce cellular S-nitrosylation, ie, CysNO and cytokine treatments, and identified 391 proteins (CysNO data set) and 313 proteins (cytokine data set) as potential SNO substrates. In all, we identified over 500 substrate proteins, most of them represent novel SNO targets, as revealed by comparison with publicly available nitrosoproteomic data. Results of biotin-switch assays validated the proteomic results for several proteins, namely galectin-1, NEDD4 and serpin B6. It should be noted that the two sets of experimental conditions correspond to very different cellular states, that is, of transient versus sustained NO/SNO formation. The treatments also differ in their effects on cell viability. Distinct from CysNO treatment, cytokine stimulation is associated with a marked decrease in the viability of A549 cells. Nonetheless, we observed that iNOS inhibition did not affect cytokine-induced toxicity. This finding appears to suggest that NO does not regulate cell death under these conditions. Nonetheless, it still remains possible that NO exerts multiple and complex effects on cell death/survival in this model. As such, this issue requires further investigation.

Bioinformatic analysis of the proteomic data showed that the identified SNO targets include proteins involved in a large number of cellular processes, including metabolism, redox homeostasis and cellular signaling. KEGG pathway enrichment analysis indicated multiple overrepresented pathways in either the CysNO or the cytokine data sets. Of interest, the number of enriched pathways and the degree of enrichment were greater in the cytokine data set relative to CysNO, which could be related to the higher specificity in target nitrosylation mediated by iNOS relative to that induced by NO donor [42]. The pathway enrichment analysis implies tight regulation by S-nitrosylation of key metabolic and signaling pathways implicated in lung inflammation and tumorigenesis, such as the pentose phosphate pathway [43] and Notch signaling [44]. Moreover, both KEGG pathway and STRING interaction analysis pointed to the cell cycle as a potentially important pathway that is regulated by S-nitrosylation. Noteworthy, among several cell cycle-related proteins that we identified as SNO targets in A549 cells was cyclin-dependent kinase 2 (CDK2). A previous study in human leukemic HL-60 cells suggested a link between the effects of NO on cell cycle progression and nitrosylation of CDK2 [45].

One aim of this study was to extract information on nitrosylation sites. Coupling of the SNO trapping approach with differential thiol labeling and MS analysis enabled the identification of nearly 100 cysteines (CysNO data set) and about 200 cysteines (cytokine data set) as putative nitrosylation sites. Comparative analysis with dbSNO supported the validity of our approach for SNO site identification. The present results notwithstanding, it should be emphasized that the current methodology, which is based on enrichment at the protein level, provides only a limited coverage of SNO sites. Comprehensive mapping of SNO sites will require further improvements of the trapping approach, including use of peptide-level enrichment.

The Trx/TrxR system constitutes a major cellular SNO reductase system [25, 46–48]. By virtue of the SNO trapping approach used in this study, it is likely that many of the identified proteins are S-nitrosylation targets of Trx. As such, our proteomic results significantly expand current knowledge regarding the Trx-regulated nitrosoproteome. The results thus point to potential cellular processes that could be modulated by Trx-mediated denitrosylation in lung cancer cells. In this regard, it is important to note that prior research has demonstrated that Trx and/or TrxR are overexpressed in various human tumors including lung cancer [49–51]. In particular, Trx was found to be widely expressed in non-small cell lung carcinomas. Trx expression in these tumors is commonly associated with high proliferation index and inversely correlates with apoptosis [52, 53]. Of further note, it was shown that A549 cells display enhanced TrxR activity and high resistance to nitrosative stress [54, 55]. These previous observations together with the present study suggest the possibility that elevated activity of Trx/TrxR could promote lung cancer progression through their denitrosylase activity. In this respect, increased denitrosylation activity could maintain key signaling proteins in their denitrosylated active state. For example, we identified the antiapototic protein XIAP as potential SNO substrate of Trx (S1 Table). As nitrosylation of XIAP is inhibitory [56], denitrosylation by Trx is expected to promote anti-apoptotic signaling, rendering the cancer cells resistant to chemotherapy or radiation [57]. More generally, it is conceivable that elevated Trx/TrxR activity could shield lung tumor cells from nitrosative stress experienced during their interaction with innate immune cells such as macrophages or in response to NO-based therapy [9, 58, 59].

In conclusion, this study represents the first analysis of the nitrosoproteome of a lung cancer cell. Our results lay the groundwork for future in-depth and detailed investigations of SNO-based regulation of individual proteins related to lung cancer progression.

## Supporting Information

S1 Fig Effect of LPS/cytokine exposure on NO production and cytotoxicity in A549 cells. (A) A549 cells were either left untreated or stimulated with LPS plus cytokines as detailed in Fig 1C. After 24, 48 and 72 h, the culture medium was assayed for nitrite accumulation (a measure of the NO released into the medium) using the Griess reaction. (B) A549 cells were left untreated or stimulated for 72 h with LPS plus cytokines in the presence or absence of 1400W (200 μM). NO production was assessed by Griess reaction. Cytotoxicity was determined using LDH assay. Results shown represent mean ± SD (n = 3).
(TIF)

S1 Table Proteomics and bioinformatics analyses. Sheets A-F list the Protein Group output from MaxQuant software and the protein ratios between the different samples based on semi-quantitative analysis (see “Materials and Methods”). Sheet G lists nitrosylated proteins identified in this study that were previously identified in the dbSNO database. Sheet H reports the comparison between SNO sites identified in this study with SNO sites previously identified in the dbSNO database. Sheet I reports the KEGG enrichment analysis of proteins categorized as candidate nitrosylated targets in the CysNO or cytokine data sets.
(XLSX)
