# Correction: Nitrosothiol-Trapping-Based Proteomic Analysis of S-Nitrosylation in Human Lung Carcinoma Cells

**DOI:** 10.1371/journal.pone.0179803

**Published:** 2017-06-12

**Authors:** Shani Ben-Lulu, Tamar Ziv, Pnina Weisman-Shomer, Moran Benhar

The concentration unit for LPS is listed incorrectly in the third sentence under the subheading “Cell culture and treatment” in the Materials and Methods section. The correct sentence is: For this purpose, cells were serum starved for 24 h and then stimulated for 72 h with or without a cytokine mix that included lipopolysaccharide (LPS, 0.5 μg/ml), tumor necrosis factor (TNFα, 20 ng/ml), interferon-γ (IFN-γ, 10 ng/ml) and interleukin 1β (IL-1β, 10 ng/ml).

The concentration unit for LPS in the caption for Fig 1C is also incorrect. Please see the corrected Fig 1 caption here.

**Fig 1:**
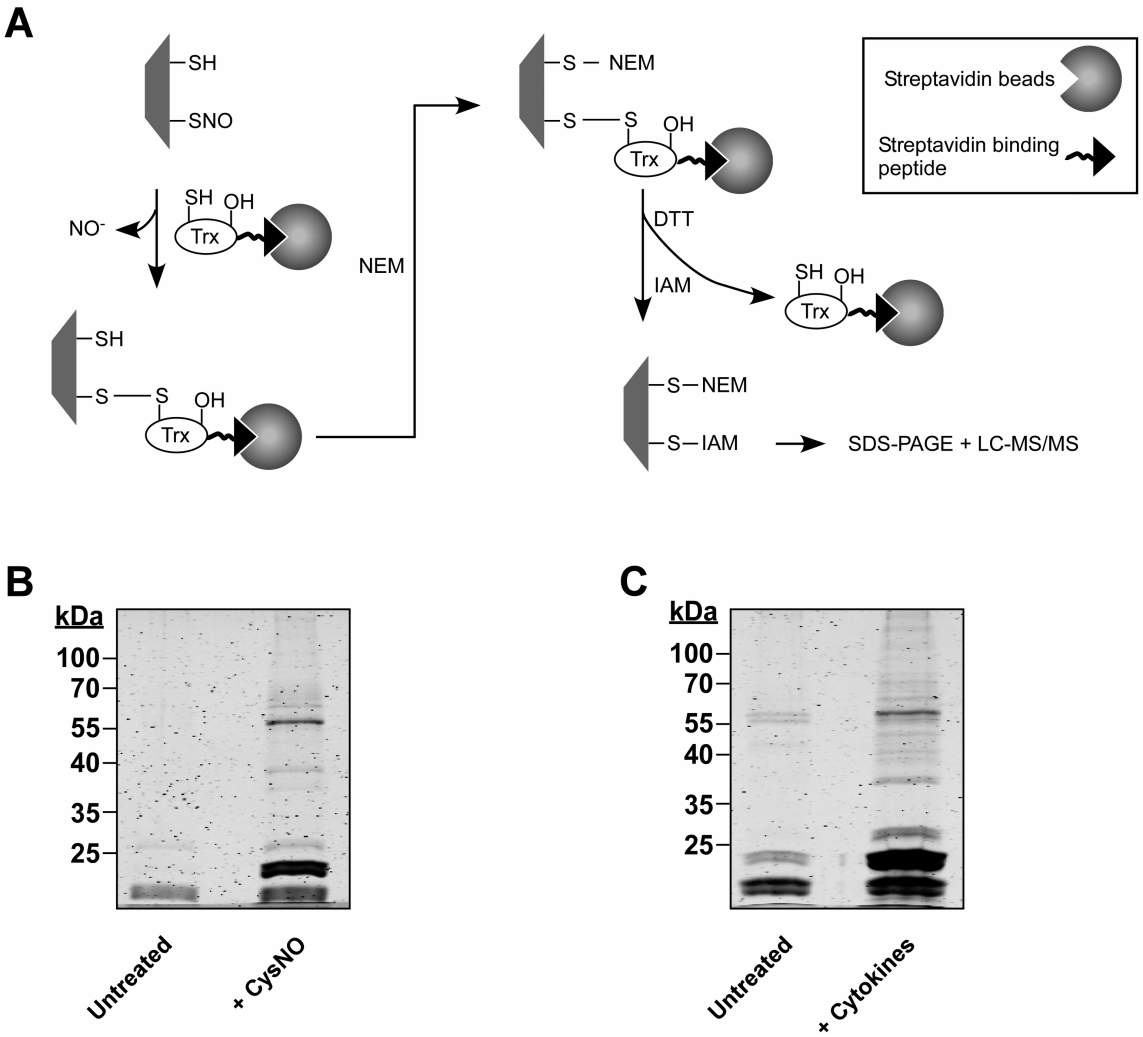
SNO trapping-based analysis of S-Nitrosylation in A549 cells. (A) Schematic of the proteomic approach. Digitonin cell lysates, obtained from A549 treated with NO donor or with cytokines are incubated with a thioredoxin (Trx) trap mutant, Trx(C35S). In the trap mutant the resolving cysteine is replaced by serine (-OH). The protein also contains a streptavidin binding peptide. Trx(C35S) forms mixed disulfide bonds with nitrosylated substrates and the resulting complexes are pulled-down using avidin agarose. Identification of nitrosylation sites is assisted by differential thiol labeling, involving the sequential application of N-ethylmaleimide (NEM) and iodoacetamide (IAM). Proteins captured in the Trx pull-down are analyzed by SDS-PAGE or liquid chromatography-tandem mass spectrometry (LC-MS/MS). (B) A549 cells were treated with or without 500 μM S-nitrosocysteine (CysNO) for 10 min and thereafter digitonin lysates were incubated with Trx(C35S). Proteins captured by Trx were released by DTT and then analyzed by SDS-PAGE. Gels were stained with Krypton fluorescent protein stain and visualized using the Odyssey infrared imaging system. (C) A549 cells were treated for 72 h with LPS (0.5 μg/ml) and a cytokine mixture that included TNFα (20 ng/ml), IFN-γ (10 ng/ml) and IL-1β (10 ng/ml). Trx-based trapping of nitrosylated proteins was performed as in B.
